# Impact of COVID-19 pandemic on autism spectrum disorder service providers in Qatar: challenges, insights, and lessons learned

**DOI:** 10.3389/fpsyt.2026.1813238

**Published:** 2026-05-05

**Authors:** Fouad Alshaban, Iman Ghazal, Fatema Al-Faraj, Sarah Aqel, Sanaa T. Al-Harahsheh, Mustafa Lotfy, Hawraa Al-Shammari, I. Richard Thompson, Assal Nasir, Mohamed Tolefat

**Affiliations:** 1 Neurological Disorders Research Center, Qatar Biomedical Research Institute, Hamad Bin Khalifa University, Qatar Foundation, Doha, Qatar; 2 College of Health and Life Sciences, Hamad Bin Khalifa University, Qatar Foundation, Doha, Qatar; 3 World Innovation Summit for Health (WISH), Qatar Foundation, Doha, Qatar; 4 Community Outreach, Qatar Autism Family Association, Doha, Qatar; 5 North East and Yorkshire NHS Genomic Laboratory Hub, England, United Kingdom; 6 Department of Biological Sciences, Drexel University, Philadelphia, PA, United States; 7 Autism Department, Shafallah Center for Children with Disabilities, Doha, Qatar

**Keywords:** ASD, COVID-19, service providers, social restrictions, stress, support

## Abstract

**Purpose:**

The COVID-19 pandemic disrupted essential services, posing unique challenges for individuals with Autism Spectrum Disorder (ASD) who depend on consistent, specialized support. Service providers faced unique challenges in adapting to remote delivery methods, highlighting the fragility of existing systems during crises. This study explored the experiences of ASD service providers in Qatar during the COVID-19 pandemic.

**Methods:**

An online survey of 66 ASD service providers in Qatar was conducted. Data were analyzed using descriptive statistics, chi-square, and likelihood ratio tests, with qualitative responses assessed through thematic analysis.

**Results:**

Most service providers (90.9%) worked remotely during the pandemic, with 81.8% engaging in online services. Providers reported significant skill regression in individuals with ASD. Stress levels were notably high (42.4%) and significantly associated with emotional tolls [p = 0.017, LR = 4.887], financial strains [p = 0.008, LR = 4.337], and personal challenges [p = 0.008, LR = 3.203]. Thematic analysis revealed decreased therapy effectiveness and difficulties in balancing work with family responsibilities.

**Conclusion:**

These findings suggest the importance of adaptive service delivery systems that maintain continuity of care during crises. Strengthening autism service infrastructure and developing resilient models are essential to safeguard autism support for future emergencies.

## 1 Introduction

The COVID-19 pandemic brought about unprecedented disruptions to every aspect of daily life, with profound implications for healthcare, education, and social systems (1). These disruptions were particularly impactful for vulnerable populations, such as individuals with Autism Spectrum Disorder (ASD), who depend heavily on consistent, specialized support to manage their developmental, behavioral, and social needs (2, 3). For many autistic individuals, this meant the abrupt cessation of therapies and interventions critical for their development and well-being (4, 5). The measures introduced to curb the spread of the virus, such as lockdowns, social distancing, and the closure of schools and healthcare facilities, led to severe interruptions in the provision of critical services for autistic individuals, as the pandemic disrupted many of these services, forcing families to delay diagnostic evaluations and consequently diminishing the effectiveness of interventions, given that early diagnosis is closely tied to positive lifelong outcomes for autistic individuals (Boyd et al., 2010; Elder et al., 2017; Warren et al., 2011). Providing intervention during the critical developmental period before the age of four leads to notable advancements in cognitive abilities, communication skills, and social interactions, while also fostering adaptive behaviors and enhancing the overall well-being of both the child and their family (6, 7). ASD-specific therapies often require direct interaction and tailored sensory engagement, all of which were severely interrupted during the pandemic. Families and caregivers were left to manage these challenges independently, often with little guidance or support (8). Research conducted in Saudi Arabia revealed that parents of autistic children experienced heightened levels of stress and anxiety while managing their care responsibilities during the COVID-19 pandemic (9).

Without consistent interventions, many faced significant regressions in behavior and skills, underscoring the importance of accessible and adaptable service delivery systems during crises (10). Research from various parts of the world has highlighted the extent of these challenges. In the United States, a study revealed that parents reported increased behavioral challenges in their children during the pandemic, with many observing a regression in previously acquired skills (11). Similarly, research in Qatar, reported that parents faced significant challenges in managing their children’s behavioral regulation, with over half experiencing deteriorations in behavior and acquired skills due to the pandemic (12). The rapid transition to telehealth and virtual platforms during the COVID-19 pandemic presented significant challenges for individuals with ASD. While Telehealth aimed to replace in-person services, they often failed to meet the complex needs of this population. These platforms frequently proved less effective and satisfactory than traditional in-person services, highlighting the importance of developing flexible and equitable care systems (13, 14). Virtual interventions lacked the tactile and sensory engagement crucial for many with ASD, rendering them less effective (15, 16). For instance, therapies requiring physical interaction, modeling, or sensory stimulation proved challenging to deliver virtually (17). Furthermore, the shift to remote learning highlighted significant disparities, as families from lower socioeconomic backgrounds struggled to provide the necessary technological resources and supportive environments for effective engagement (18).

Service providers play a critical role in supporting the developmental, social, and behavioral needs of individuals with ASD, often bridging the gap between families and essential resources. During the COVID-19 pandemic, the rapid transition to remote service delivery introduced a myriad of challenges. Many providers reported difficulty adapting interventions that traditionally required hands-on or sensory-based approaches to virtual formats, leading to concerns about the efficacy of these services (19). Additionally, technological limitations, including inadequate access to devices and low technological literacy among families, further impeded service delivery (20). Service providers, too, faced challenges in adapting to new technologies, often feeling unprepared to deliver effective care in virtual formats. These obstacles, compounded by the pressure to meet the needs of families in a rapidly changing environment, took a significant toll on their emotional and mental well-being (21, 22). Uncertainty surrounding evolving public health guidelines, coupled with concerns about the long-term impact of service disruptions on individuals with ASD, created additional stress (23). Providers noted feelings of helplessness when unable to respond effectively to crises, such as behavioral outbursts, through virtual means (19). Despite their central role, the experiences of ASD service providers during the pandemic remain underexplored (24). This gap in the literature is critical, as providers were often the primary source of guidance and support for families navigating the pandemic’s challenges (25).

In Qatar, where considerable advancements have been made in expanding ASD services, the disruptions caused by the pandemic highlight the need to examine the experiences of service providers who play a critical role in supporting autistic individuals. While previous research has focused on the impact of COVID-19 on families of children with ASD, the perspectives and challenges faced by service providers remain underexplored. This study represents the second phase of our comprehensive research on the effects of the COVID-19 pandemic on ASD in Qatar. Phase 1 explored the pandemic’s impact on families of individuals diagnosed with ASD (12). Building on this foundation, the current study evaluates the experiences of ASD service providers in Qatar during the COVID-19 pandemic, highlighting key barriers, adaptations, and lessons learned to enhance future resilience in service delivery. By focusing on this unique context, the findings aim to contribute to global discussions on strengthening ASD services during times of crisis.

## 2 Materials and methods

### 2.1 Study design

To assess the impact of COVID-19 control measures on service providers in Qatar who support individuals with autism and their families, a mixed-methods approach was adopted. This involved collecting both quantitative and qualitative survey data. A customized questionnaire was developed and distributed via Google Forms for data collection. The survey was conducted between March and June 2021. Participants provided informed consent electronically through the platform or via email. The study received ethical approval from Qatar Biomedical Research Institute Institutional Review Board (IRB) (QBRI-IRB 2020-07-023). All procedures adhered to the ethical standards of the institutional and/or national research committees, as well as the Helsinki Declaration and its subsequent amendments or equivalent ethical guidelines.

### 2.2 Participants

A convenience sample of 66 ASD service providers in Qatar was utilized for this study. These participants represented various institutions, including hospitals, autism centers, special needs centers, and schools dedicated to supporting individuals with Autism. The participants held diverse specialties such as occupational therapists (OTs), speech and language therapists (SLTs), Psychologists, Physicians, Pharmacists, Nurses, Teachers, Physiotherapists, and Rehabilitation specialists. Recruitment involved contacting professionals through institutional databases, email invitations, and direct outreach. No formal sample size calculation was conducted, as recruitment was based on participant availability.

### 2.3 Instruments

An online survey targeting service providers was created using Google Forms and disseminated through a hyperlink. Participants provided electronic informed consent, which included detailed information about the study’s purpose, procedures, benefits, voluntary participation, and researcher contact details.

Given the absence of a standardized instrument specifically designed to assess the impact of COVID-19 on ASD service providers, a study-specific questionnaire was developed by the research team. The development process was informed by existing frameworks, including the Autism Parenting Stress Index (APSI) and the WHO Quality of Life (WHOQOL), which guided the conceptual domains covered in the questionnaire, such as stress, service disruption, emotional impact, financial strain, and professional challenges.

The questionnaire consisted of 25 items combining multiple-choice and open-ended questions. To assess clarity, relevance, and face validity, the survey was pilot tested with three ASD service providers. Feedback from the pilot focused on clarity of wording, appropriateness of response options, and completion time, and minor revisions were made accordingly.

It is important to note that the questionnaire was not formally validated as a psychometric tool and was used for exploratory purposes in this study.

To accommodate the linguistic preferences of participants, the questionnaire and consent form were translated into Arabic by bilingual researchers. Translation accuracy was reviewed by bilingual team members to ensure consistency of meaning. Among the final respondents, 47 (71.2%) completed the survey in English, and 19 (28.8%) in Arabic. The final version of the survey included 25 questions, consisting of both multiple-choice and open-ended formats. These questions addressed topics such as sociodemographic information, the impact of the pandemic on professional roles, access to services and resources, challenges encountered during the pandemic, and the overall well-being of service providers.

### 2.4 Procedures

The initial version of the questionnaire underwent a thorough revision process involving three authors (FA, SA, and IG), ensuring its relevance and clarity. All researchers involved in data collection were bilingual Arab professionals with extensive experience in autism research within the Arab community. Two distinct recruitment methods were utilized to engage participants.

First, service providers were invited via email, which included a link to the survey. Those who agreed to participate completed and submitted the survey electronically. Second, participants were contacted by phone, where the purpose of the study was explained. Providers were then given the option to complete the survey over the phone with a researcher or independently. In all cases, consent was obtained electronically through an online form. Responses from phone interviews were recorded directly by researchers into the survey form.

This dual recruitment strategy was particularly appropriate given the context of the study, which coincided with the COVID-19 pandemic and related social distancing measures, leading to the temporary closure of centers, schools, and clinics. Completing the survey required approximately 10–15 minutes. The questions focused on the challenges faced by service providers during the pandemic, particularly the period of confinement and physical distancing in 2020.

### 2.5 Data analysis

The final dataset was exported from Google Forms into a Microsoft Excel file and analyzed using SPSS software (Version 26.0). Descriptive statistics were applied to summarize the sample characteristics and survey responses. Chi-square and likelihood ratio tests were conducted to examine the relationship between stress experienced by ASD service providers and key variables, including remote work, providing in-home therapy during lockdown, skills regression in ASD individuals, engagement in online or additional ASD services, financial strains, emotional tolls, personal challenges, and health impact.

To further explore the challenges faced by service providers during the pandemic, qualitative data collected through semi-structured forms was analyzed using thematic content analysis following established qualitative frameworks (26). Themes and subthemes were identified and supported with illustrative examples. Three authors (FA, SA, and IG) independently categorized responses into different themes. Any discrepancies in categorization were resolved through discussion to achieve consensus.

Three authors (FA, SA, and IG) independently reviewed the responses and conducted initial coding. Codes were then grouped into broader categories and themes through an iterative process. The researchers met to compare coding, discuss discrepancies, and reach consensus on final themes.

Responses submitted in Arabic were translated into English by bilingual members of the research team prior to analysis, ensuring preservation of meaning and context. Quotations originally submitted in Arabic were translated into English for reporting by bilingual members of the research team.

## 3 Results

### 3.1 Sociodemographic information

Service providers reported their demographic and professional characteristics, including gender, nationality, marital status, employment status, and work arrangements prior to the lockdowns. Among the participants, 54.5% were female and 45.5% were male. The majority of participants (80.3%) were Arab, while the remaining 19.7% were from non-Arab countries. 72.7% of the participants were therapists, including occupational therapists, speech and language therapists, physiotherapists, and rehabilitation specialists. 18.2% were teachers, comprising special education teachers, parent trainers, and principals. 9.1% were medical providers, including physicians, psychologists, nurses, and pharmacists. Regarding workplace distribution, most participants worked in governmental sectors (95.5%) while others were employed in private sectors (4.5%). All participants were employed at the time of survey. Before the lockdown, the majority of service providers worked full-time (87.9%), while a smaller proportion (12.1%) worked part-time (see **Table 1** for details).

**Table 1:** Sociodemographic characteristics of the participants.

Variable	Category	n	%
Gender	Male	30	45.5%
	Female	36	54.5%
Nationality	Arabs	53	80.3%
	Other countries	13	19.7%
Employment Status	Employed	66	100%
Work Status Before Lockdowns	Full-time	58	87.9%
	Part-time	8	12.1%
Position	Therapists	48	72.7%
	Medical providers	6	9.1%
	Teachers	12	18.2%
Employment Sector	Governmental	63	95.5%
	Private	3	4.5%

### 3.2 Survey responses: quantitative analysis of survey results

#### 3.2.1 Service adaptations during the COVID-19 pandemic for ASD individuals

The study examined the impact of the COVID-19 pandemic on service provision for individuals with autism spectrum disorder (ASD) (**Table 2**). A significant proportion of participants (90.9%) reported working from home during the pandemic. In-home therapy saw notable changes, with 22.7% providing such services before the pandemic and 39.4% continuing during the pandemic. Interestingly, 19.7% of service providers started offering in-home therapy for the first time during the pandemic. Online service provision was highly utilized, with 81.8% of participants engaging in this modality. When explaining COVID-19 preventative measures to individuals with ASD, service providers predominantly relied on visual aids, with 69.7% using pictures and 47.0% employing verbal explanations. Social stories were used by 37.9%, while 16.7% of providers reported being unable to explain these measures effectively. A smaller group (19.6%) used other methods. Skill regression among individuals with ASD under care was also reported. A majority noticed regression to some extent (43.9%), while 47.0% observed a more pronounced regression in previously gained skills. These findings underscore the significant adaptations made by ASD service providers during the pandemic and the challenges faced in maintaining skill levels among individuals with ASD.

**Table 2:** Changes in ASD service delivery and skill regression during the pandemic.

Survey question	Yes (N)	%
Did you work from home during the pandemic?	60	90.90%
Were you providing in-home therapy before COVID-19?	15	22.70%
Did you provide in-home therapy during COVID-19?	26	39.40%
Did you start in-home therapy for the first time during pandemic?	13	19.70%
Did you engage in online service provision?	54	81.80%
How were COVID-19 preventative measures explained to individuals with ASD under your care?		
Verbally	31	47.00%
With Pictures	46	69.70%
Social Story	25	37.90%
I Couldn’t Explain	11	16.70%
Others	13	19.60%
Since the start of the home quarantine, did you notice any regression in the individuals’ previously gained skills?		
To some extent	29	43.90%
Yes	31	47.00%

Percentages may exceed 100% due to multiple responses.

#### 3.2.2 Confidence, stress, and impact of pandemic-related measures on service providers

Service providers rated their confidence and readiness for online service provision on a scale from 1 to 5. Nearly half (48.5%) felt neutral about their readiness, while 21.2% expressed confidence, and another 21.2% reported feeling very confident. A smaller proportion felt slightly confident (7.6%), and only 1.5% reported no confidence at all. Regarding stress levels during this period, the majority of providers reported elevated stress. Over 42.4% described themselves as stressed, with an additional 12.1% feeling very stressed. About 31.8% remained neutral, while smaller percentages reported having normal levels of stress (7.6%) or not stressed at all (6.1%). The pandemic also negatively affected service providers in various ways. The majority (84.8%) reported being emotionally impacted, and 57.6% faced personal challenges. Financial strain was experienced by 40.9% of providers, while 36.4% reported health-related impacts. These findings emphasize the multifaceted challenges faced by service providers during the pandemic, affecting their confidence, stress levels, and overall well-being. See **Table 3** for details. Significant associations were observed between stress and financial strain [χ² (1, N = 66) = 7.028, p = 0.008], emotional toll [χ² (1, N = 66) = 5.673, p = 0.017], and personal challenges [χ² (1, N = 66) = 6.956, p = 0.008] (**Table 4**). Emotional toll showed the strongest association with higher reported stress levels (Likelihood Ratio = 4.887), followed by financial strain (Likelihood Ratio = 4.337) and personal challenges (Likelihood Ratio = 3.203). Other service-related variables, including in-home therapy [χ² (1, N = 66) = 0.171, p = 0.679] and online ASD services [χ² (1, N = 66) = 0.122, p = 0.727], were not significantly associated with stress levels. However, providing additional ASD services during lockdown showed a marginal association [χ² (1, N = 66) = 3.564, p = 0.059], suggesting a potential relationship worth further investigation.

**Table 3:** Confidence, stress, and impact of pandemic-related measures on service providers.

Survey question	N	%
On a scale of 1-5, how confident and ready did you feel about the online service provision?		
Not Confident	1	1.50%
Normal level of confidence	5	7.60%
Neutral	32	48.50%
Confident	14	21.20%
Very Confident	14	21.20%
On a scale of 1-5, how would you rate your stress level during this time?		
Not Stressed at All	4	6.10%
Normal level of stress	5	7.60%
Neutral	21	31.80%
Stressed	28	42.40%
Very Stressed	8	12.10%
Kindly tick all that applies to you: the pandemic related measures have negatively affected me:		
Financially	27	40.90%
Emotionally	56	84.80%
Personally	38	57.60%
Healthy wise	24	36.40%

**Table 4:** Assessment of service provider stress using chi-square and likelihood ratio.

Variable	Pearson’s χ²	Likelihood ratio
Remote work during lockdown	1.265 (N = 66, df = 2, p = 0.531)	1.615
In-Home therapy during lockdown	0.171 (N = 66, df = 1, p = 0.679)	2.521
Skills regression in ASD individuals	0.055 (N = 66, df = 1, p = 0.815)	0.134
Online ASD Services	0.122 (N = 66, df = 1, p = 0.727)	0.118
Providing additional ASD services	3.564 (N = 66, df = 1, p = 0.059)	6.165
Financial Strain	7.028 (N = 66, df = 1, p = 0.008)	4.337
Emotional Toll	5.673 (N = 66, df = 1, p = 0.017)	4.887
Personal challenges	6.956 (N = 66, df = 1, p = 0.008)	3.203
Health Impact	0.963 (N = 66, df = 1, p = 0.327)	0.876

### 3.3 Qualitative analysis: impact of COVID-19 lockdowns on in-home therapy and service providers

#### 3.3.1 Impact on in-home therapy services

Thematic analysis revealed significant challenges and adaptations in in-home therapy services during the pandemic (**Table 5**). A major theme was the negative impact on service provision, with 45.5% of participants highlighting reduced therapy effectiveness and skill deterioration. Providers emphasized that online therapy was not as effective as face-to-face sessions, leading to a decline in children’s progress, as one participant noted, “Therapy sessions online are not as effective as face-to-face.” The pandemic also had emotional and behavioral effects on children, reported by 27.3% of participants. Anxiety, regression in skills, and a lack of socialization were commonly observed, with one provider stating, “Lack of socialization has caused anxiety among ASD children.” Providers also reported service quality and accessibility challenges (22.7%). Reduced quality of online sessions and limited access to resources made therapy less effective. A participant noted, “Limited access to resources and inconsistent service provision negatively affected therapeutic goals.” Despite these challenges, some service providers noted positive adaptations and coping strategies (18.2%). Many families became more involved in therapy, with parents taking an active role in their children’s learning. A participant shared, “Families learned how to help their children during the pandemic.”

**Table 5:** Impact of COVID-19 lockdowns on in-home therapy services.

Theme	Subtheme	n	%	Example quotations
Negative Impacts on Service Provision	Reduced therapy effectiveness, skill deterioration	30	45.5%	*“Therapy sessions online are not as effective as face-to-face.”* *“ Skills gained by children are deteriorating due to lack of direct intervention.”* *“Children are not getting enough services due to restrictions.”*
Positive Adaptations and Coping Strategies	Parents taking an active role in therapy	12	18.2%	*“Some parents became more involved in their children’s learning.”* *“ Parents are more hands-on, which is a good change for Qatar.”* *“ Families learned how to help their children during the pandemic.”*
Emotional and Behavioral Effects on Children	Anxiety, regression in skills, and lack of socialization	18	27.3%	*“Many children experienced regression in communication and behavioral skills.”* *“Lack of socialization has caused anxiety among ASD children.”* *“Children’s emotional and social well-being is significantly affected.”*
Service Quality and Accessibility Challenges	Lower quality of online sessions and reduced access	15	22.7%	*“Online therapy is less effective and difficult to implement.”* *“ Limited access to resources and inconsistent service provision.”* *“Sessions were disrupted, negatively affecting therapeutic goals.”*

Percentages may exceed 100% due to multiple responses.

#### 3.3.2 Challenges and adaptations of service providers

Service providers faced multiple challenges due to pandemic-related measures (**Table 6**), including emotional challenges (37.9%). Stress, isolation, and uncertainty were common, as one provider described, “I felt isolated and scared that I might never go back to my previous life”. Balancing work and family responsibilities was another significant issue, with work-life balance challenges affecting 22.7% of participants. One provider shared, “Balancing work and children’s online learning was extremely challenging”. Financial strain, reported by 18.2% of participants, was also a concern. Salary cuts, unpaid leave, and increased expenses added pressure, as one provider said, “New expenses like masks, sanitizers, and delivery services strained our budget.” Health concerns, such as fear of infection and physical health impacts, were highlighted by 15.2% of participants. One provider shared, “Stress from being exposed to potential infection daily was overwhelming.”

**Table 6:** Challenges and adaptations of service providers due to pandemic-related measures.

Theme	Subtheme	n	%	Example quotations
Emotional Challenges	Stress, isolation, and uncertainty	25	37.9%	*“Felt isolated, always thought: am I doing my best to support my students?”* *“I felt sad and scared that I might never go back to my previous life.”*
Work-Life Balance Issues	Managing family and work responsibilities	15	22.7%	*“Balancing work and children online learning was extremely challenging.”* *“I had to neglect my own children due to work demands.”* *“Being a mother of 3 boys who switched to online learning made it hard to attend to both work and family.”*
Financial Strain	Salary cuts and additional expenses	12	18.2%	*“Salary cuts and unpaid leave impacted us financially.”* *“New expenses like masks, sanitizers, and delivery services strained our budget.”* *“Uncertainty of income and financial security was stressful.”*
Health Concerns	Fear of infection and health impacts	10	15.2%	*“Fear of contracting COVID-19 and spreading it to family members.”* *“Concerns about health security were overwhelming.”* *“Stress from being exposed to potential infection daily.”* *“Health-wise, the pandemic caused fear and a decline in physical and emotional well-being.”*
Service Delivery Challenges	Changes in therapy and communication	18	27.3%	*“Providing therapy remotely was less effective.”* *“Changes in service delivery and communication with families were difficult to manage.”* *“Dealing with cases of ASD required direct intervention, which was hindered during the pandemic.”* *“Challenges adapting to new therapeutic strategies.”*
Adaptation and Positivity	Creativity and resilience	8	12.1%	*“The pandemic taught me to be more creative in my sessions.”* *“I started researching ways to improve therapy and make sessions more effective.”* *“It made me unselfish, thinking about others’ needs while adapting to new challenges.”* *“I tried new strategies to face these challenges and help my students.”*

Percentages may exceed 100% due to multiple responses.

Service delivery challenges, affecting 27.3% of participants, were another prominent theme. Remote therapy was viewed as less effective, with one provider noting, “Dealing with cases of ASD required direct intervention, which was hindered during the pandemic”. Amid these challenges, some providers demonstrated adaptation and positivity (12.1%), finding ways to be creative and resilient. As one participant remarked, “The pandemic taught me to be more creative in my sessions.”

#### 3.3.3 Suggestions for improving ASD services for future pandemics

Service providers offered various suggestions to improve ASD services during pandemic situations, focusing on empowering families, enhancing service flexibility, and addressing physical and emotional well-being (**Table 7**). The most frequently cited suggestion was parental empowerment through training and guidance (30.3%). Providers highlighted the importance of educating caregivers on therapeutic strategies, increasing family participation in therapy, and establishing consistent routines at home. One provider emphasized, “Empowering parents with quality education on strategies used”. Improving service delivery through hybrid and flexible models was another common theme (22.7%). Participants recommended combining online and in-person sessions, deploying mobile teams for home care, and providing one-on-one therapy with proper safety precautions. As one provider suggested, “Use a blended model for special education schools.” Technology and accessibility improvements were also highlighted (18.2%), with providers suggesting enhancements to teletherapy tools, creating video tutorials, and improving families’ technology skills. A participant noted, “Provide more digital resources for therapy.” Promoting physical and emotional well-being was another key recommendation (15.2%), with suggestions such as outdoor activities, reducing screen time, and focusing on family-based engagement to alleviate stress. One provider shared, “Focus on mental health instead of academic goals.” Community and policy support was also identified as important (12.1%), with suggestions for fostering collaboration between public and private sectors, utilizing outdoor spaces like parks for therapy, and raising awareness through family engagement programs. As one participant noted, “Develop long-term strategies for similar crises.”

**Table 7:** Suggestions for improving ASD services during a pandemic.

Theme	Subtheme	n	%	Example quotations
Parental Empowerment	Training and guidance	20	30.30%	*“Empowering parents with quality education on strategies used.”*
				*“Provide caregiver training on therapeutic goals.”*
				*“Increase family participation in therapy.”*
				*“Focus on creating consistent routines at home.”*
Service Delivery Improvements	Hybrid and flexible models	15	22.70%	*“Use a blended model for special education schools.”*
				*“Offer online and in-person sessions.”*
				*“Mobile teams for home care to ensure service continuity.”*
				*“Provide one-on-one therapy with proper precautions.”*
Technology Use	Tools and accessibility	12	18.20%	*“Improve online applications and teletherapy tools.”*
				*“Create video tutorials for therapy implementation.”*
				*“Enhance computer and technology communication skills for families.”*
				*“Provide more digital resources for therapy.”*
Physical and Emotional Wellbeing	Outdoor and exercise-based activities	10	15.20%	*“Open outdoor play areas for ASD children.”*
				*“Encourage physical activities to reduce stress.”*
				*“Decrease screen time and involve children in family-based activities.”*
				*“Focus on mental health instead of academic goals.”*
Community and Policy Support	Awareness and collaboration	8	12.10%	*“Increase collaboration between public and private sectors.”*
				*“Use public parks and outdoor spaces for therapy.”*
				*“Enhance family awareness programs to improve engagement.”*
				*“Develop long-term strategies for similar crises.”*

Percentages may exceed 100% due to multiple responses.

## 4 Discussion

The COVID-19 pandemic has significantly impacted service delivery for individuals with ASD, necessitating rapid adaptations by service providers. A substantial majority transitioned to remote work, with many adopting online platforms to continue providing care. This shift aligns with global trends, as telehealth became a primary mode of service delivery during the pandemic. Such shifts have been widely recognized for their ability to ensure continuity of care but have also been associated with challenges in engagement, particularly for individuals with ASD who often struggle with virtual communication environments (27). Despite these efforts, challenges persisted. Many individuals with ASD experienced regression in previously acquired skills, highlighting the limitations of remote interventions. This regression highlights the critical need for maintaining continuity of care and the potential benefits of hybrid service delivery models that combine in-person and virtual elements (28). The pandemic also exacerbated existing disparities in service access, particularly among marginalized communities. Studies have shown that disruptions in services disproportionately affected families from lower socioeconomic backgrounds, emphasizing the need for equitable service delivery strategies (29). The COVID-19 pandemic significantly disrupted in-home therapy services for children with ASD, as evidenced by our thematic analysis. A substantial portion of service providers (45.5%) reported diminished therapy effectiveness and skill regression, attributing these issues to the limitations of online therapy compared to traditional face-to-face sessions. This observation is consistent with what Ferguson et al. found, where caregivers perceived behavioral therapy services delivered via telehealth as less effective and more difficult to engage with, resulting in lower satisfaction (13). Additionally, 27.3% of providers observed increased anxiety and decreased socialization among children during the pandemic. These observations are consistent with research highlighting the adverse psychological impacts of the pandemic on individuals with ASD, including heightened anxiety and behavioral challenges (24). Challenges in service quality and accessibility were noted by 22.7% of participants, who pointed to limited resources and inconsistent service provision as barriers to effective therapy. This aligns with previous findings which reported that the COVID-19 pandemic negatively impacted the social communication and interaction of children and adolescents with autism spectrum disorder, highlighting the challenges in maintaining consistent and effective therapeutic services during this period (30).

A significant portion of providers reported elevated stress levels, with 42.4% feeling stressed and 12.1% very stressed. This is consistent with reports indicating that approximately half of healthcare workers experienced burnout amid the pandemic (31). The emotional and financial impacts reported by providers in our study are also echoed in the literature. A study on the mental health impact of the pandemic on healthcare workers found significant emotional tolls, with many experiencing psychological distress (32). Financial strain was also a notable concern, particularly for providers working in private centers, as the pandemic disrupted traditional service delivery models, leading to a loss of income and increased financial uncertainty. Furthermore, our analysis revealed significant associations between stress levels and factors such as financial strain, emotional toll, and personal challenges. These findings are in line with research highlighting that stress among healthcare personnel is influenced by multiple factors, including personal and professional challenges during the pandemic (33). Moreover, 22.7% of participants reported difficulties in managing professional duties with personal responsibilities, similar to findings from studies showing that the pandemic intensified these issues (34). Despite these challenges, some participants demonstrated adaptation and positivity, finding creative ways to continue service provision. This resilience is reflected in literature that show how healthcare professionals adapted to unprecedented circumstances by developing new strategies to deliver care effectively (33).

The qualitative insights from service providers highlighted the necessity for adaptive strategies in delivering ASD services during pandemic situations. A significant emphasis was placed on empowering parents through targeted training and guidance, highlighting the importance of educating caregivers on therapeutic strategies and increasing family participation in therapy. This approach aligns with existing literature that underscores the effectiveness of parent-mediated interventions in enhancing outcomes for children with ASD. For instance, research found that training parents in specific therapeutic techniques led to significant improvements in children’s social communication skills (27). Additionally, service providers recommended the adoption of hybrid and flexible service delivery models, combining online and in-person sessions to ensure continuity of care. Enhancing technology and accessibility was also highlighted, with providers advocating for improvements in teletherapy tools and digital resources. Studies have emphasized the need for accessible and user-friendly technological solutions to facilitate effective remote interventions for individuals with ASD (35). Furthermore, promoting physical and emotional well-being through outdoor activities and focusing on mental health was deemed essential. The pandemic has negatively impacted the mental health of individuals with ASD, emphasizing the need for interventions that address both physical and emotional well-being (36). Lastly, fostering community and policy support through collaboration between public and private sectors and developing long-term strategies was identified as crucial. The Interagency Autism Coordinating Committee has emphasized the importance of coordinated efforts to support the autism community during public health emergencies (Interagency Autism Coordinating Committee, n.d).

## 5 Limitations

This study has several limitations that should be considered when interpreting the findings. First, the use of a convenience sample limits the generalizability of the results to all ASD service providers in Qatar. The sample was predominantly drawn from the public sector, which may underrepresent the experiences of professionals working in private settings.

Second, the relatively small sample size (n = 66), while adequate for exploratory analysis, limits the ability to conduct more advanced statistical modeling and may reduce statistical power.

Third, the questionnaire used in this study was not formally validated as a psychometric instrument. Although it was pilot tested for clarity and relevance, the lack of formal validation may affect the reliability and interpretation of some measures.

Additionally, data were collected between March and June 2021, and some questions required participants to reflect on earlier stages of the pandemic, introducing the possibility of recall bias.

Despite these limitations, the study provides valuable exploratory insights that contribute to the emerging understanding of ASD service provision during crisis situations.

## 6 Conclusion

The COVID-19 pandemic and associated lockdown measures caused widespread disruptions globally, disproportionately affecting vulnerable populations, including individuals with ASD. The sudden shift in service delivery, challenges in maintaining therapeutic routines, and increased stress levels among both families and service providers highlighted potential vulnerabilities in existing service systems.

While service providers demonstrated resilience and adaptability during the pandemic, the challenges encountered underscore the importance of developing more flexible and responsive service delivery models. These findings suggest that such models may help support continuity of care during future crises and improve accessibility for individuals with ASD.

This study also highlights the potential value of empowering families through targeted training and engagement, as well as the use of hybrid service modalities that combine in-person and virtual interventions. Additionally, attention to the physical and emotional well-being of both individuals with ASD and service providers appears important for sustaining effective care delivery.

Overall, these findings contribute to the growing body of evidence on ASD service provision during public health emergencies and may help inform future research and service planning aimed at enhancing resilience and inclusivity within ASD care systems.

## Data Availability

The raw data supporting the conclusions of this article will be made available by the authors, without undue reservation.
