# Supplementary material for: Impact of COVID-19 pandemic on autism spectrum disorder service providers in Qatar: challenges, insights, and lessons learned
Source: Front Psychiatry. 2026 May 5;17:1813238. doi: 10.3389/fpsyt.2026.1813238 (PMC13183810; doi:10.3389/fpsyt.2026.1813238)
Supplement: Supplementary file 2 [file DataSheet2.pdf]

SURVEY TO MEASURE THE EFFECT OF COVID-19 PANDEMIC ON SERVICE PROVIDERS FOR INDIVIDUALS WITH ASD IN QATAR

This questionnaire was developed by the research team to assess the impact of COVID-19 on ASD service providers in Qatar. The development was informed by conceptual domains derived from the Autism Parenting Stress Index (APSI) and the WHO Quality of Life (WHOQOL). The questionnaire was pilot tested for clarity and relevance but was not formally validated as a psychometric tool.

The questionnaire is presented as administered to participants, with only minor formatting adjustments for clarity.

There is no doubt that the worldwide pandemic of COVID-19 has affected nearly all aspects of life, including health and safety of all people, especially individuals with special needs like those affected by Autism Spectrum Disorder.

Measures taken by most countries to control and limit the spread of this pandemic including Qatar, with the closure of schools and centers providing treatment, rehabilitation, and educational services for individuals with ASD and training services for ASD providers, has affected all students and individuals with ASD in particular, and the ASD service provision in general.

In light of the current situation, Qatar Biomedical Research Institute (QBRI), the Qatar Autism Families Association (QAFA), and the World Innovation Summit for Health (WISH) have taken an initiative to support the families of individuals with ASD and service providers to enable them to express their concerns and struggles during the period of the COVID-19 pandemic.

Hence, we request from all individuals involved in the service provision of individuals with ASD to participate in this survey to share their experience with the current situation of school and center closures, which led to the suspension of all direct intervention services, as well as overall drastic changes in ASD service provision.

Section A: Demographic Information

1. **This study has been approved by the QBRI Institutional Review Board (ethics committee). If you wish to participate, kindly check the box that applies to you: ***
   - Yes
   - No
2. **Nationality: ____**
3. **Name of institution: _________________________________**
4. **Position/specialty: __________________________________**
5. **Gender ***
   - Male
   - Female

6. **Marital status:**
    - Single
    - Married
7. **Employment status ***
    - Employed
    - Not Employed
    - Self-employed

Section B: Employment and Service Provision During COVID-19

8. **During the peak of the COVID-19 situation, did you work from home? ***
    - Yes
    - No
    - N/A - unemployed
9. **Prior to the COVID-19 related lockdowns, were you working:**
    - Full-time
    - Part-time
10. **Were you providing in-home therapy prior to COVID-19? ***
    - Yes
    - No
11. **Did you continue to provide in-home therapy during COVID-19 lockdown?**
    - Yes
    - No
12. **Did the COVID-19 related lockdown affect your in-home therapy service, please explain:**

    ____

    ____

13. **Did you provide additional services in other centers/schools prior to COVID-19?**
    - Yes
    - No
14. **Did you continue to provide additional services in other centers/school during COVID-19 lockdown?**
    - Yes
    - No
15. **Did you engage in online service provision for individuals with ASD?**
    - Yes
    - No

Section C: Confidence and Service Adaptation

16. **How confident and ready did you feel about the online service provision?**
    1 = Not confident
    2 = Slightly confident
    3 = Neutral
    4 = Confident
    5 = Very confident

17. How were COVID-19 preventative measures explained to individuals with ASD under your care? *

- VERBALLY
- WITH PICTURES
- SOCIAL STORY
- I COULDN'T EXPLAIN
- Other:____

18. Since the start of the home quarantine, did you notice any regression in the individuals' previously gained skills? *

- Yes
- No
- To some extent

Section D: Stress and Impact

19. On a scale of 1-5, how would you rate your stress level during this time: *

1 = Not stressed at all
2 = Slightly stressed
3 = Neutral
4 = Stressed
5 = Very stressed

20. Kindly tick all that applies to you: the pandemic related measures have negatively affected me:

- Financially
- Emotionally
- Personally
- Health-wise

21. Kindly elaborate on the previous question of how you feel the pandemic related measures have affected you, the main challenges you are facing, etc. *

Section E: Open-Ended Questions

22. As a professional in the field of intervention for individuals with ASD, what do you suggest could have been done the same or differently to ameliorate the negative effects of the pandemic related measures on individuals with ASD and their families, as well as on the service providers?

23. As the duration of this pandemic is yet unknown, how do you perceive this affecting the ASD service provision in general?

24. What do you suggest could be improved in terms of service provision for individuals with ASD during a pandemic?

25. Kindly provide us with your preferred mode of communication; phone number/email, if you don't wish to be contacted, write "no":
